# The effect of a parental preparation video (Take5) on child and parent anxiety during anaesthetic induction: a protocol for a randomised controlled trial

**DOI:** 10.1186/s13063-023-07480-0

**Published:** 2023-07-08

**Authors:** Krittika Vongkiatkajorn, Erin A. Brown, Alexandra Donaldson, Vanessa Rich, Rebecca Paterson, Justin Kenardy, Cameron Graydon, Paul Lee-Archer

**Affiliations:** 1 Anaesthetics Department, Queensland Children’s Hospital, Queensland Health, South Brisbane, Australia; 2 Child Health Research Centre, School of Medicine, The University of Queensland, Brisbane, Australia; 3 Melbourne School of Psychological Sciences, University of Melbourne, Melbourne, Australia; 4 School of Psychology, The University of Queensland, Jamieson Trauma Institute, Royal Brisbane and Women’s Hospital, Queensland Health, Brisbane, Australia

**Keywords:** Peri-operative anxiety, Anaesthetic induction, Randomised controlled trial, Paediatric, Procedural anxiety, Psychoeducation intervention, Parent–child relationship, Parenting behaviour

## Abstract

**Background:**

Children undergoing anaesthetic induction experience peri-operative anxiety associated with negative outcomes including emergence delirium, short- and long-term maladaptive behaviour and increased postoperative analgesic requirements. This stems from children’s limited ability to communicate, cope, and regulate intense emotions, leading to high dependency on parental emotional regulation. Previous interventions including video modelling, education and distraction techniques before and during anaesthetic induction have demonstrated significant reduction of anxiety levels. No existing interventions combines evidenced-based psychoeducation video with distraction techniques to support parents to moderate peri-operative anxiety. This study aims to test the efficacy of the Take5 video (now referred to as ‘Take5’), a short and cost-efficient intervention for child peri-operative anxiety.

**Methods:**

A randomised, controlled, superiority trial of Take5 compared to standard care. Take5 was developed by paediatric anaesthetists, child psychologists and a consumer panel of parents of children who had experienced surgery and anaesthesia.

Children aged 3–10 years presenting for elective surgery at a quaternary paediatric facility will be randomly allocated to the intervention group or standard care. Intervention group parents will be shown Take5 prior to accompanying their child for anaesthesia induction. Primary outcomes include child and parent anxiety at induction, measured by the Modified Yale Preoperative Anxiety Scale Short Form (mYPAS-SF), the Peri-operative Adult–Child Behavior Interaction Scale (PACBIS) and the Induction Compliance Checklist (ICC). Secondary outcomes include post-operative pain, emergence delirium, parental satisfaction, cost-effectiveness, parent and child psychological well-being at 3 months post procedure and video intervention acceptability.

**Discussion:**

Perioperative anxiety is associated with negative outcome in children including higher pharmacological intervention, delayed procedures, and poor post-recovery outcomes resulting in financial burden on health systems. Current strategies minimising paediatric procedural distress are resource-intensive and have been inconsistent in reducing anxiety and negative postoperative outcomes. The Take5 video is an evidence-driven resource that is designed to prepare and empower parents. The success of Take5 will be evaluated by measuring differences in patient (acute and 3-month), family (satisfaction, acceptability), clinician (feasibility) and health service (cost) outcomes, with each anticipated to benefit children.

**Trial registration:**

Australian and New Zealand Clinical Trial Registry (ACTRN12621001337864) and Children’s Health Queensland Hospital and Health Service Human Research Ethics Committee (HREC/21/QCHQ/73894).

**Supplementary Information:**

The online version contains supplementary material available at 10.1186/s13063-023-07480-0.

## Background and rationale

Research reports 65% to 78% of children experience significant levels of peri-operative anxiety [1–3]. Acute levels of anxiety and distress are associated to psychological issues including mood disorders: depression, anxiety and oppositional defiant disorder and phobias [4], physical impacts such as eating and sleeping disturbances and enuresis [5], and developmental issues notwithstanding cognitive decline and poor development of toileting, independence and maturing of emotional regulation skills [6]. Peri-operative anxiety has also shown to activate physiological responses (cortisol and catecholamines, antibody production and cytokine secretion) that lead to short- and long-term negative outcomes [1]. The most immediate symptoms associated are emergence delirium, expected in 12–18% of all children undergoing anaesthetic induction [7], higher levels of post-operative pain [8, 9], increased analgesic use post-discharge [8] and slower recovery [3, 9]. It has been reported these negative outcomes are experienced by 54% of children within the first 2 weeks, with ongoing negative consequences at 6 (27%) and 12 months (7%) post-surgery [5, 10, 11]. While most children are resilient, approximately 10–13% will experience chronic negative outcomes prolonging physical recovery [4, 12, 13].

At present, pre-operative anxiety is managed through either pharmacological (pre-medication) and/or non-pharmacological intervention. Pharmacological interventions include administering medications that reduce pre-operative anxiety but this can be associated with risk of post-traumatic stress symptoms, increased costs, hospital delays, slower discharge from hospital [3] and, for certain medications, such as benzodiazepine (i.e. midazolam) increased incidence of emergence delirium [14–17]. Alternatively, non-pharmacological interventions such as distraction (i.e. video games/technology), hypnotherapy, clown doctors and acupuncture are preferred due to minimal side-effects, yet evidence of their effectiveness remains variable [18]. While the types of non-pharmacological interventions are increasing, parental presence remains the most employed and studied despite mixed findings surrounding its effectiveness.

A Cochrane review which examined 28 trials, totalling 2681 children over two decades concluded parental presence had no effect on child’s levels of anxiety, distress, or improved child cooperation during anaesthetic induction [18]. However, an evidenced-based review by Chundamala [19] found mixed results, providing further insight. Specifically, a retrospective cohort analysis by Kain of 568 participants across 7 years, of children aged 2–12, found ‘calm parents’ significantly reduced anxiety among ‘anxious children’ and ‘anxious parents’ significantly increased anxiety among ‘calm children’ [20]. However, no effects were found between ‘calm parents’ to ‘calm children’ or ‘anxious parents’ to ‘anxious children’ [20]. Similarly, a randomised control trial (RCT) by Johnston [21] among 134 children aged 2–8, found that ‘anxious parents’ increased anxiety for children, whereas the presence of ‘calm parents’ made no difference whether they were present or absent during an anaesthetic induction on the child’s level of anxiety. These two studies imply it is not parental presence alone but parental emotional state that significantly influences child anxiety, particularly when there is a misalignment between child and parent emotional states. Furthermore, ceiling and floor effects were reported, such that once a certain level of anxiety or calmness was reached, parental presence with an aligned emotional state did not change the child’s emotional state. This indicates value in interventions that address parental anxiety.

Of most interest is Patel’s study of 112 children aged between 4 and 12, which found the greatest decrease in paediatric anxiety through parental presence was in conjunction with distraction, specifically when patients were provided a hand-held device (i.e. video game) by the research team [22]. The study found this combination more effective compared to parental presence alone or parental presence with the administration of midazolam [22]. That is, the inclusion of a video game provided an opportunity for parents to engage in coping-promoting behaviour towards their child. Supporting the use of distraction are two systematic reviews that found game-based interventions (i.e. gamification or virtual reality) during paediatric anaesthetic induction were effective in reducing anxiety for the child and parent [23, 24]. This finding suggests that parent emotional states can be influenced and is further enhanced with distraction, an important consideration to maximising the impact of parental presence.

A key reason why parental presence is heavily relied upon despite a lack of evidence on its effectiveness is the significant association between the parent’s and the child’s emotional regulation. Specifically, children are dependent on their parents to moderate their emotions, and theory-driven research supports that parental procedural behaviour mediates parental psychological distress and child procedural distress [1, 25]. An important barrier, however, to the impact of parental presence on peri-operative anxiety is parental anxiety. Specifically, parents can also experience ongoing distress from a child’s medical procedure and associated illness/injury [11]. Approximately 25% of parents report heightened anxiety and traumatic stress symptomology in the first month, and 5% have ongoing symptoms at 6 months [25]. Consequently due to the parent’s own anxiety, their ability to support their child’s emotional regulation is diminished [21]. That parents also experience distress provides a potential explanation for the conclusion of the Cochrane review that parental presence alone was ineffective in reducing child’s anxiety [18]. This relationship between parent and child emotions is the theoretical foundation of the current study. As parents prefer to be present during anaesthetic induction [26], this study targets two key levers intervening the parent’s emotional state and equipping parents with coping-promoting behaviours including distraction strategies, with the overall goal of reducing child anxiety.

Presently, two seminal studies aimed at intervening negative parental presence using video preparation and modelling, have found evidence for a concomitant and significant reduction in peri-operative anxiety among children. One study evaluated the ADVANCE program, a multicomponent behavioural program targeting anxiety reduction through distraction, video modelling, education and coaching parents regarding positive and negative coping behaviours [14]. Overall, the program was effective at significantly reducing peri-operative anxiety among children during anaesthetic induction compared to pre-medication, parental presence and standard hospital care groups [14]. These children also had significantly lower incidences of emergence delirium, required less analgesia and demonstrated faster recovery times [14]. A major limitation of the ADVANCE program, however, is the limited feasibility and application to many hospital settings, as it required extensive lead up time and intensive parental coaching [27].

The second study by Bailey and colleagues, tested a brief video intervention on 93 parents of children aged 2–10 years, supplied on the day of the surgery [27]. The video provided information about the procedure, addressed parental anxiety and explained the benefits of distraction strategies. Parents in the intervention group tended to report higher self-efficacy regarding their role during the induction [27]. This is important as parents have identified that it was important for them to be present to support their child’s procedures and this increase in self-efficacy would like increase their confidence and lower their levels of anxiety [26, 27]. Nevertheless, no statistical differences were found between groups on child peri-operative anxiety behaviour, emergence delirium, postoperative pain, post operative analgesia, or recovery time. A significant limitation of this study was that it did not measure parental behaviour to determine whether the intervention was successful in changing parental behaviour, the key mediator of child procedural anxiety [28].

It is important to assess accessibility, mode, and timing of delivery to understand contributing factors to effectively administer a video intervention. Presently, evidence-based video interventions like ADVANCE [14] by Kain and colleagues for perioperative anxiety are resource intensive and time-consuming and is unlikely to be feasible in most health care settings [27]. Conversely, brief video interventions, particularly those that address parental anxiety, are a promising non-pharmacological intervention but are currently under-evaluated. Lastly, there is a lack of understanding of how parents consolidate information under a stressful environment (i.e. peri-operative) and limited identification of the potential enablers and barriers to parents maintaining a calm state.

Based on theory-driven research [28, 29], Brown and colleagues developed and feasibility tested the Take5 video intervention, designed for parents of children undergoing burn wound care. Initial results indicated lower rates of distress and pain for children of parents who had received the video, compared to an observational cohort (unpublished). This intervention was the foundation for the current Take5 intervention for parents of children undergoing anaesthetic induction. A group of professionals, in consultation with a parent consumer group, forward-developed Take5 for anaesthetics to address the current gaps in the literature. Specifically, a representative parent consumer group informed the appropriateness of language, tone, and sensitivity required in communicating to parents in anxious states based on lived experiences. Furthermore, the Take5 intervention was tested for acceptability with 10 families attending elective surgery prior to data collection for further refinement of the intervention. The end product was a 5-min animated video that aimed to provide parents with procedural preparation, behavioural coaching to engage the child in distraction, and psychological self-coping strategies based on the evidence-based Acceptance Commitment Therapy Framework [30].

The intervention will be tested against a control group which will receive standard of care consisting of preoperative preparation provided by the anaesthetic doctors including a description of the anaesthesia management and a discussion of the risks involved.

### Objectives

The study aims to test the efficacy of the Take5 video intervention at reducing child and parent anxiety with the following key objectives:

1. Demonstrate the efficacy of the ‘Take5’ resource in reducing preoperative anxiety and postoperative adverse outcomes via a RCT at a quaternary paediatric facility.
2. Demonstrate the efficacy of ‘Take5’ in reducing adverse psychological and health-related quality of life 3 months post-surgery, as well as improving parental satisfaction of care.
3. Demonstrate the health economic benefits of the ‘Take5’ resource.

### Hypotheses

Based on its prior initial success, it is expected that a ‘Take5’ video tailored to the peri-operative context will demonstrate significant reductions in peri-operative child anxiety and improve post-operative outcomes. Specifically, it is hypothesised:

1. Children in the Take5 intervention group will demonstrate less peri-operative distress behaviours than children in the control group.
2. Children in the Take5 intervention group will report less peri-operative anxiety than children in the control group.
3. Parents in the Take5 intervention group will report less peri-operative anxiety than parents in the control group.

Secondary hypotheses are:

1. Children of parents in the intervention condition will experience lower rates of post-operative pain scores and emergence delirium scores, and faster time to discharge compared to children in the control group.
2. Children in the Take5 intervention will have improved health-related quality of life scores and reduced behavioural difficulties including symptoms of anxiety and depression at 3 months after the surgery compared to children in the control group.
3. Parents in the Take5 intervention will report less general anxiety and depressive symptoms at 3 months after their child’s surgery compared to parents in the control group.

### Trial design

This single-centre, randomised controlled efficacy trial with two parallel arms is comparing the clinical effectiveness and cost-effectiveness of two approaches to reducing peri-operative anxiety in children:

1. Standard care: preoperative preparation provided by the anaesthetic team including a description of the anaesthesia management and a discussion of the risks involved.
2. Take5: a brief video provided to parents prior to their child undergoing anaesthetic induction.

The RCT is reported in accordance with the Standard Protocol Items: Recommendations for Intervention Trials (SPIRIT) 2013 statement for clinical trial protocols [31].

## Methods: participants, interventions, and outcomes

### Study setting

This trial began recruitment on 22 June 2022 and is expected to continue recruitment until March 2023. The study is based at a quaternary paediatric facility in Queensland, Australia. The facility admits approximately 6500 children (neonates to 17 years old) annually for elective procedures [32].

### Eligibility criteria

Eligible participants are patients aged between 3 and 10 (inclusive) years old undergoing elective day-case procedures at a quaternary paediatric facility who do not meet the following exclusion criteria. Exclusion criteria consist of children (1) with a diagnosed pervasive developmental disorder or global developmental delay documented in their medical records, (2) requiring emergency surgery, (3) under the care of the Department of Child Safety, (4) who have a parent with insufficient English to provide consent without the aid of a translator, (5) deemed by anaesthetist as requiring pre-medication (i.e. midazolam) prior to a general anaesthetic, and (6) deemed highly anxious by the anaesthetist who will not benefit from additional personnel.

### Interventions

Take5 was designed to be implemented as a universal tool for all parents who attend their child’s medical procedure, to increase parental psychological coping and associated behaviours, to target the child’s procedural coping. Given the acute delivery design, Take5 was intentionally brief to be provided via a tablet in a clinical environment on the day of surgery prior to the procedure.

Specifically, Take5 communicated three key messages: What to expect during the patient journey (procedural preparation), the benefits of distraction to the child (reinforcing parental behaviour), and psychoeducation to provide general coping strategies for the parent to manage personal distress during and after the child’s procedure. Procedural preparation was communicated because patients and parents commonly express unmet expectations for information [33]. Positive behavioural reinforcement was used because fear-based messaging is thought to have unintentional consequences [34]. The psychoeducation was modelled on Acceptance Commitment Therapy to assist with normalising feelings of distress for parents [22]. An acceptability and feasibility study (unpublished) of 10 parents receiving Take5 demonstrated good acceptability, and increased positive parenting behaviours, and reduced child procedural distress, compared to data from Brown et al.’s observational study [28].

The ‘Take5’ video was adapted by the authors (a group of psychologists and anaesthesiologists) for anaesthetic induction. The authors initially consulted the hospital’s consumer panel of parents to understand what preparatory information parents valued regarding the anaesthetic induction, what helped their child have a positive medical experience, and what advice they had for parents to cope while supporting their child. The panel feedback was documented, thematically summarised, and agreed by the authors as in or out of scope for the video. In-scope feedback pertained to the key messages and generalisability to other hospital locations. Using the in-scope panel feedback, the authors adapted the procedural preparation, parental behaviour and psychoeducation components of Take5 to ensure appropriate for anaesthetic induction. The authors presented a drafted video script back to the consumer panel for validation and further feedback. The panel provided positive comments on the script and suggestions for the animation.

The video was developed using a VideoScribe web subscription, and images were chosen to minimise gender, ethnicity, and language biases. Further parental feedback was elicited through 10 data collection ‘dry runs’, with small changes made to the video prior to commencing recruitment.

### Outcomes

The primary outcome of the study is child peri-operative anxiety as measured by the modified Yale Preoperative Anxiety Scale Short Form (mYPAS-SF) [35], the Peri-operative Adult–Child Behavior Interaction Scale (PACBIS) [36], the Visual Analogue Scale for Anxiety (VAS-A) [37], and the Induction Compliance Checklist (ICC) [38].

The secondary outcomes include:

1. Child post-operative pain measured during recovery via observation using the Face, Legs, Activity, Cry and Consolability Pain Scale (FLACC) [39];
2. Child emergence delirium scored during recovery using the Cornell Assessment for Paediatric Delirium (CAP-D) [40];
3. Parent satisfaction with procedure via a purpose-built survey of feedback via a numeric rating scale (1–10 of increasing satisfaction);
4. Parent and child 3-month psychosocial outcomes: measured using validated measures of adult psychological well-being, specifically the Depression, Anxiety and Stress Scale (DASS-21) [41], child physical, mental and social health the Patient-Reported Outcomes Measurement Information System (PROMIS [42] and PROMIS Early Childhood [43]) and health-related quality of life using the Child Health Utility instrument (CHU9D) [44];
5. Acceptability: A semi-structured interview developed in specifically for the ‘Take5’ video will assess acceptability of delivery and content of ‘Take5’; and
6. Cost-effectiveness via the cost of intervention, and direct and indirect healthcare costs to the healthcare system.

### Participant timeline

Enrolment and consenting of participants occur when parents and child (patient) arrive on their day of surgery, have been placed in the holding bay and are deemed eligible for the study. The holding bay is where patients wait after being admitted into the hospital, and receive a medical check by nurses and doctors to ensure they are fit and ready for surgery. This pre-operative environment is a busy clinical setting and parents are approached after all clinical checks have been conducted and the anaesthetist has confirmed to the research team whether pre-medication will be given (part of exclusion criteria). Parents of patients who will not receive pre-medication are approached for recruitment.

Time period 1 (t1) is defined as time at the holding bay after the family has been enrolled in the study and allocated to a study condition. At this time, the intervention group are the provided with the Take5 video in addition to standard care. This will occur approximately 15 min prior to surgery. Comparatively, the control will only experience standard care. The anaesthetist and research team aim to provide sufficient time for parents to watch the video and process the information prior to anaesthetic induction. Parents of both groups are asked their levels of anxiety measured by the VAS-A, however, parents in the intervention group will be asked after watching the Take5 video. The child’s anxiety will be observed by the researcher using the mYPAS-SF scale and parent-reported measured by the VAS-A. Parent and child behaviours at this time are observed measured by the PACBIS. When the surgical team are ready, the parent and child will be escorted from the holding bay into the induction theatre.

Time period 2 (t2) is defined as from when the parent and child is inside the induction theatre and when the anaesthetist commences the induction procedure, until the child is fully anaesthetised. Procedural commencement may involve the anaesthetist engaging the child with non-procedural talk, helping the child get on the bed, offering the mask to the child or beginning the intravenous preparation. This time period is expected to be 3–10 min in length. During this time, video recording and capturing of observation data occurs, including parent–child behaviours measured by the PACBIS, child’s anxiety measured by the mYPAS-SF, and child’s cooperation measured by the ICC. After the child is fully anaesthetised and the parent leaves the theatre, the parent will be asked to rate their own and child's level of anxiety during the induction, as well as their current (post-induction) level of anxiety, measured by the VAS-A.

Time period 3 (t3) is defined as when the child regains consciousness (general anaesthesia wears off) after the procedure. At this point, clinical staff will score the child for emergence delirium measured by the CAP-D and pain measured by the FLACC.

Time period 4 (t4) is defined as approximately day 92 (i.e. 3 months post discharge) after the child’s surgery where the participating parent will be asked to self-report anxiety and depression symptoms measured by the DASS-21 and provide a proxy-report for the child's physical, mental and social health measured by the PROMIS and health-related quality of life measured by the CHU9D.

### Sample size

The primary outcome of this study is children’s anxiety at anaesthesia induction as measured by the mYPAS-SF. Our estimate of effect is based on the validation study of ‘Take5’ in a cohort of children with burn-injuries (unpublished) and the ADVANCE preparation program [14]. Assuming a clinically meaningful difference in anxiety (15-point difference on the mYPAS-SF) [8, 14], with a mean control group score = 55 and a mean intervention group score = 40, a corresponding effect size of 0.61 was used for these analyses. Therefore, a sample of 50 participants per group is anticipated to provide > 90% power to detect this effect size (α < 0.05).

### Recruitment

The clinical research assistant (social worker with psychology training) will perform recruitment, obtain written informed consent, provide the Take5 video intervention as per randomisation through sealed envelopes, video record the anaesthetic induction and undertake data collection. All participant data will be scored under interrater reliability by another member of the research team blinded to group allocation.

Patients who are scheduled for elective surgery will be screened for eligibility by a trained research assistant daily (weekdays) by reviewing the elective surgery list. Eligibility for the study will be confirmed with the rostered consultant anaesthetist. Participants who meet eligibility criteria will be approached to participate by the research assistant while at the Holding Bay on the day of surgery prior to receiving the anaesthetic induction. If the research assistant considers the parent to be under significant psychological distress some normalisation of the experience and follow up care will be provided to the participant as part of duty of care. A record of all patients screened exclusion criteria and reason for refusal will be recorded by the research assistant.

Recruited parents will be asked to report the levels of anxiety for themselves (parent) and their child. Parents allocated to the control group will be asked prior to the anaesthetic induction, whereas parents in the intervention group be asked after receiving the video intervention. Both groups will be filmed during their anaesthetic induction and parents will be asked to report their own and their child's anxiety levels during and after induction, and their own satisfaction level with the anaesthetic induction experience. Care will be taken to ensure the filming is unobtrusive and will not increase the child’s or parent’s anxiety. All participants will receive questionnaires after 3 months post-surgery to follow up on their mental and physical outcomes.

## Methods: assignment of interventions

### Allocation

#### Sequence generation

Numbers 1 to 100, equally identifying “intervention” and “control”, were manually allocated to individual envelopes. Once envelops were sealed, sequencing occurred through randomisation by a third party who unsystematically rearranged the sealed envelopes. The RA is blinded to the final sequence making the selection between intervention and control unpredictable.

#### Concealment mechanism

The envelopes will be prepared by a third-party separate to the trial; however, masking the research assistant, patient and their parent to the treatment allocation will not be possible.

#### Implementation

After consent is obtained, the research assistant will open a sealed envelope to determine whether participants are in the intervention and control group.

#### Blinding

Clinical staff (anaesthetic doctors and nurses) will be blinded to the treatment allocation. As induction will be filmed, the researcher responsible for coding the behavioural measures will also be blinded to treatment allocation.

## Methods: data collection, management, and analysis

### Data collection and methods

The research assistant will collect data on primary/secondary outcomes by observation, chart audit, and from staff and patient families during the peri-operative phases. To ensure inter-rater reliability and to prevent having too many people in the room when the child is having their anaesthetic, induction will be filmed allowing a second blinded investigator to score the child’s anxiety. Follow-up data will be collected using a purpose-built, online survey. A timeline of study participation and measures is summarised in Table 1.

**Table 1:** Standard Protocol Items: Recommendations for Intervention Trials (SPIRIT 2013) schedule of study recruitment, intervention, and assessments

	*Study period*					
	*Enrolment*	Allocation	*Post-allocation*			
Timepoint	* − t*_*1*_	0	*t*_*1*_	*t*_*2*_	*t*_*3*_	*t*_*4*_
Enrolment:						
Eligibility screen	X					
Informed consent	X					
Allocation		X				
Interventions:						
Take5			X			
Control (Standard Care)			X			
Assessments:						
Visual Analogue Scale—Anxiety (VAS-A)			X	X		
Modified Yale Preoperative Anxiety Scale Short Form (mYPAS-SF)			X	X		
Perioperative Adult–Child Behavior Interaction Scale (PACBIS)			X	X		
Induction Compliance Checklist (ICC)			X	X		
Cornell Assessment for Pediatric Delirium (CAP-D)					X	
Face, Legs, Activity, Cry, Consolability Scale (FLACC)					X	
Parent rated acceptability of Take5 Video (*n* = 10)				X		
Parent rated satisfaction of clinical service						X
Patient-Reported Outcome Measures Early Childhood (PROMIS EC) – Child 3–5 years						X
Patient-Reported Outcome Measures (PROMIS) – Child 6–10 years						X
Child Health Utility 9D (CHU9D)						X
Depression Anxiety and Stress Scale (DASS-21)						X

### Reliability of measures

- Modified Yale Preoperative Anxiety Scale Short Form (mYPAS-SF) (α = 0.92) [35]
- Peri-operative Adult–Child Behavior Interaction Scale (PACBIS) (Kappa range 0.62 to 0.94) [36]
- Induction Compliance Checklist (ICC) (α = 0.99) [38]
- Depression, Anxiety and Stress Scale 21 (DASS-21) (α = 0.74) [41]
- Child Physical, Mental and Social Health (Patient-Reported Outcomes Measurement Information System (PROMIS [42] and PROMIS Early Childhood [43]))
- Child Health-Related Quality of Life (Child Health Utility Instrument: CHU-9D) [44]

### Data collection plan retention

Participants are contacted twice, via a text message one week after the 3 month follow up is due and a telephone call 2 weeks later if the survey remains uncompleted. Participants choosing to withdraw from the study can do so without any repercussion and data will be destroyed. Participants identified retrospectively as meeting the exclusion criteria will be excluded from the study and additional participants will be recruited to fulfil the sample size (Table 1).

### Data management

All data will be entered into dedicated secure, online database (Research Electronic Data Capture [REDCap]) [45]. Quality checks and monitoring of all source participant data will be undertaken by the research assistant. Forms used for data collection can be found in Additional file 2: Appendix 2.

### Statistical methods

#### Statistical methods for primary and secondary outcomes

Interrater reliability will be conducted by having two researchers independently scoring every anaesthetic induction for observational measures including mYPAS-SF, PACBIS and ICC. Following training in the measures, reliability will be attained by the researchers independently scoring sets of five videos, and statistically comparing results using intra-class correlations, as recommended for ordinal data [46]. The researchers will discuss any discrepancies in scoring, before independently scoring another set of five videos, until reliability is achieved. To test for inter-rater drift, reliability was assessed at 20% intervals throughout data collection.

Between group comparisons of primary and secondary outcomes will be compared using general linear modelling, to account for relevant control variables. Secondary outcomes that are non-continuous will be analysed using Pearson’s chi-squared analysis. The primary analysis will be ‘intention-to-treat’. A within-trial cost-effectiveness analysis will be conducted. Resource consumptions (and cost) will be collected for both arms (standard and interventions). Primary outcomes of behaviour and anxiety, and the secondary outcome (quality of life) will be used. Incremental cost and effectiveness (outcomes) will be calculated, and the incremental cost per effectiveness ratio (ICER) will be the primary result. Data will be reported in accordance with the Consolidated Standards of Reporting Trials (CONSORT) 2010 statement [47].

## Methods: monitoring

### Data monitoring

Data collection occurs at specific time points and no further monitoring of data is required for the study.

### Harms

Given the non-invasive nature of the intervention, no new adverse events are expected. In general, the most common negative outcomes associated with anaesthetic induction are child distress resulting in escalation to pharmacological intervention, and/or parent/guardian distress. If heightened child and/or parent/guardian distress occurs in either arm of the trial, treatment will be in accordance with the institution’s clinical practice guidelines. It is expected that a proportion of children and/or parents/guardians will experience distress during this trial, but the rates are expected to be no higher than standard care. Child and/or parent/guardian distress is recorded as part of the feasibility data. Given the potential for this distress to persist after the completion of surgery and discharge from the hospital, a member of the research team (psychologist) will follow up with any parents/guardians to ensure access to necessary support if required. Any serious adverse events and protocol modifications will be reported to the HREC. If important protocol modifications are required (e.g. changes to eligibility criteria, outcomes or analyses), PLA will update all investigators, HRECs, information and consent forms, the trial registry and the publishing journal. Clinical trial insurance is held by the university.

## Discussion

Medical procedures, such as anaesthetic induction, can be particularly fear-inducing for the child and stressful for the parent, due to being in an unfamiliar environment, fear of pain, and high levels of uncertainty [48]. Experiences of peri-operative anxiety can also lead to ongoing procedural anxiety and medical traumatic stress [11]. Younger children have less developed skills to understand and rationalise the benefits of medical care and communicate and manage their fears. This makes children particularly at risk of procedural distress [11].

While older children can more easily engage in gamification distraction, younger children require more direct support. Parents, as the primary caregivers, are the logical point of first contact for young children to seek out support in uncertain situations such as an anaesthetic induction. Parents, therefore, can intentionally provide distraction intervention to their child to minimise child anxiety. Considering the strong relationship between parent and child anxiety [49], and the impact parental distress has on parenting behaviour during medical procedures [28], upskilling the parent as an intervention needs to ensure the parent can also manage their own distress. Generally, interventions looking to modify parental behaviour during medical procedures have not directly addressed parental distress.

The Take5 video resource, is a low impact, short and cost-effective intervention aimed at empowering and equipping parents with psychoeducation and distraction techniques to moderate paediatric peri-operative anxiety. The impact of effectively reducing peri-operative anxiety includes requiring less pharmacological intervention, minimising short and long-term negative post-recovery outcomes and reducing the overall burden on health systems.

## Trial status

Protocol version 1.0, recruited commenced in June 2022, expected completion March 2023.

## Supplementary Information

**Additional file 1.**

**Additional file 2.**

## Data Availability

Participants or the research team may request to access data pertaining to the project. Protocol amendments Protocol amendments are submitted to the Human Research Ethics Committee of our institution. Consent or assent The research assistant will seek prospective written and informed consent from the patient’s parent or guardian prior to enrolment in the study, with consent able to be withdrawn. All withdrawal data will be collected and included in the CONSORT diagram [47]. Participant data will not be identified by name. Confidentiality De-identified raw data will be stored within a data repository and made available to other researchers upon reasonable request and following review of such a request by the original ethics committees. Ancillary and post-trial care If participants experience persistent distress after the completion of surgery and discharge from the hospital, a member of the research team (Psychologist) will follow up with any parents/guardians to ensure access to necessary support if required.
