# Supplementary figures and images for: The effect of a parental preparation video (Take5) on child and parent anxiety during anaesthetic induction: a protocol for a randomised controlled trial
Source: Trials. 2023 Jul 8;24:446. doi: 10.1186/s13063-023-07480-0 (PMC10329363; doi:10.1186/s13063-023-07480-0)

#### Appendix 1

#### Data collection form


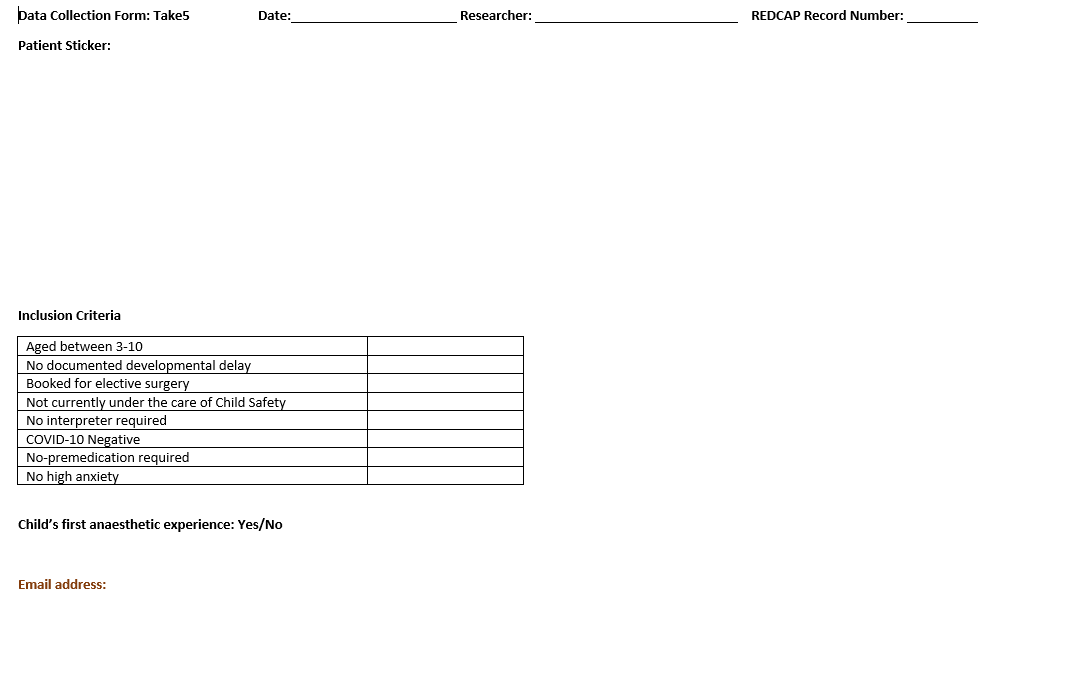


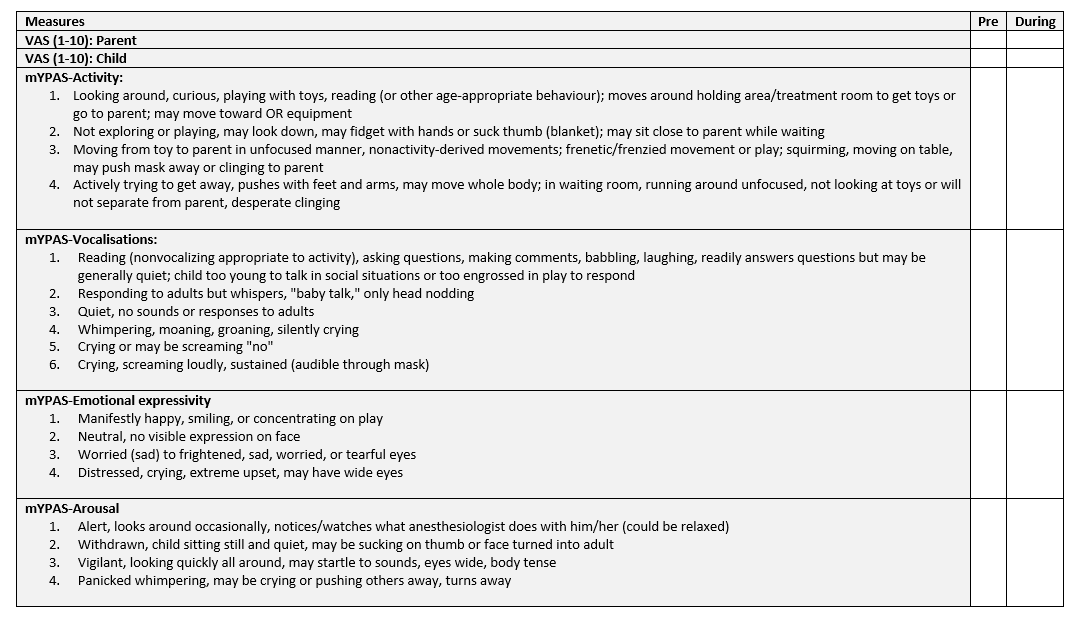


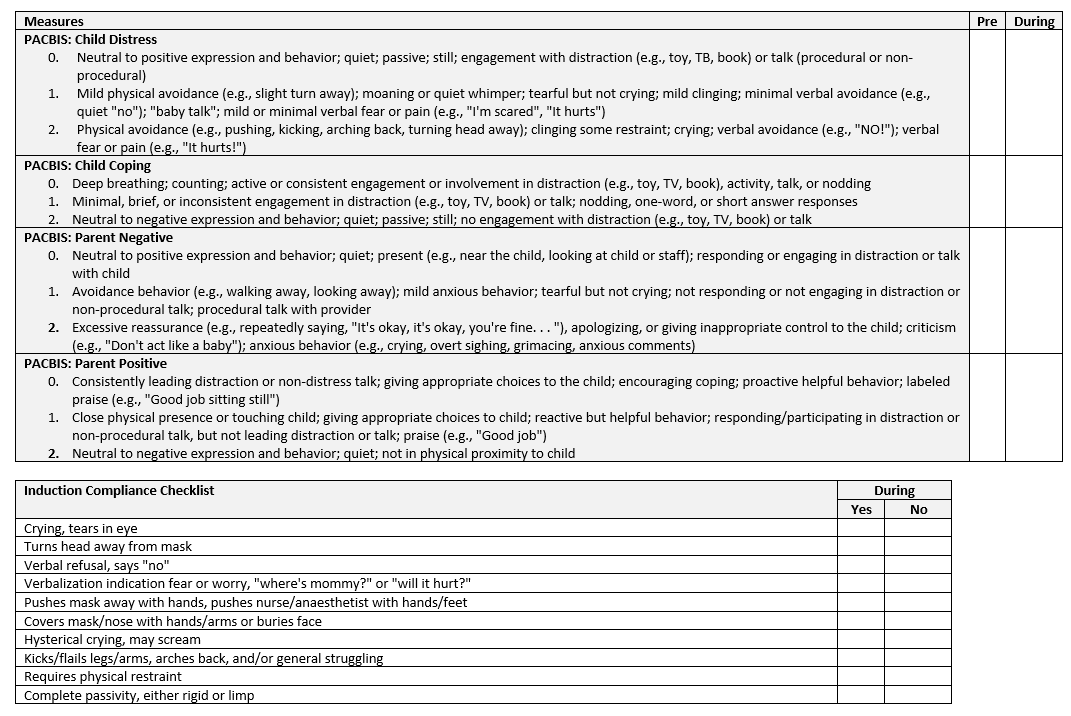


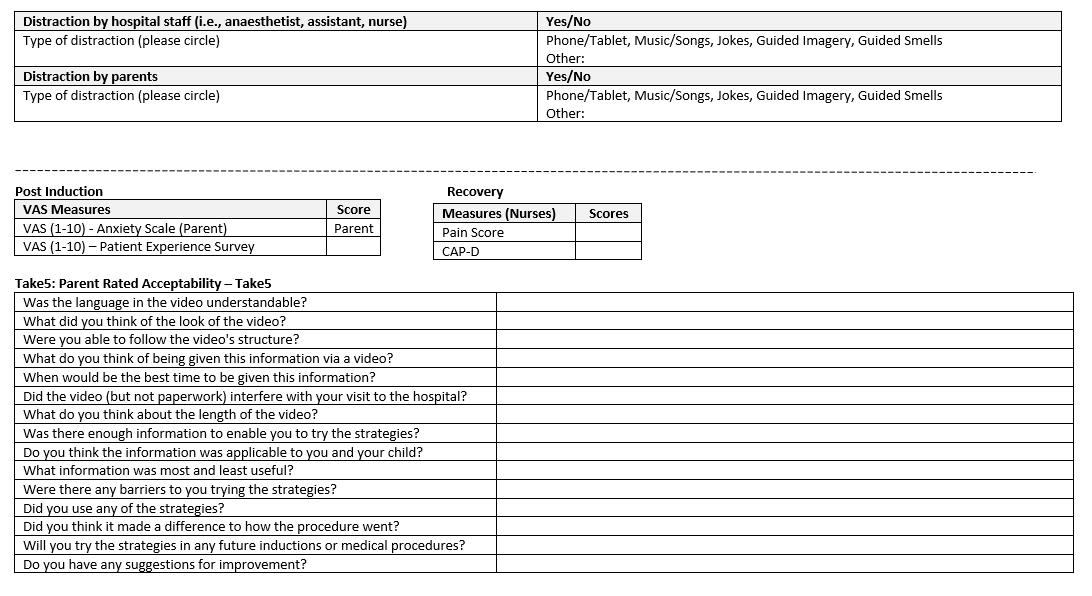

Supplement: Supplementary file 1 — Additional file 1. [file 13063_2023_7480_MOESM1_ESM.docx]

Appendix 2

Informed consent materials


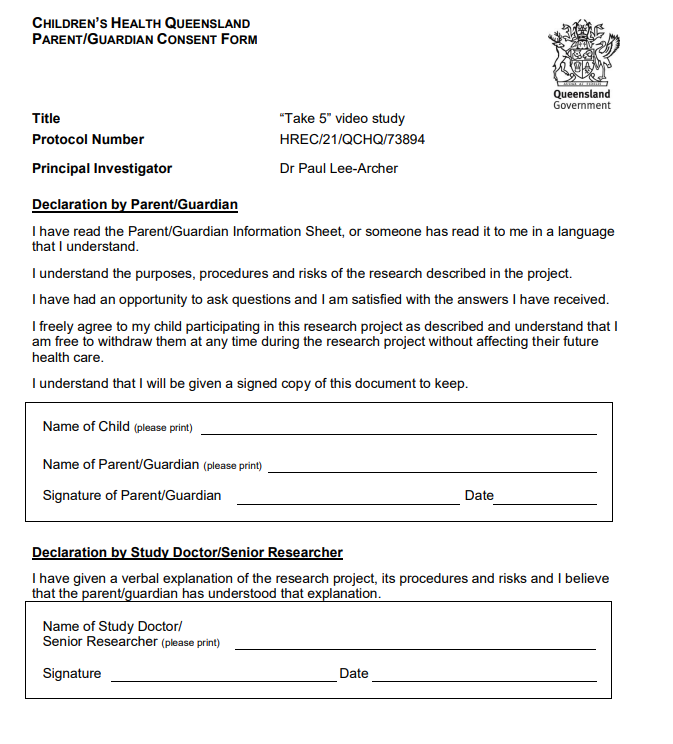

Supplement: Supplementary file 2 — Additional file 2. [file 13063_2023_7480_MOESM2_ESM.docx]
